# Supplementary material for: Heavy metals in water and sediment of Cikijing River, Rancaekek District, West Java: Contamination distribution and ecological risk assessment
Source: PLoS One. 2024 Apr 17;19(4):e0294642. doi: 10.1371/journal.pone.0294642 (PMC11023565; doi:10.1371/journal.pone.0294642)
Supplement: S1 File — (DOCX) [file pone.0294642.s001.docx]

**Table 1.** Results of Measurement of Physical-Chemical Parameters in the Rainy Season on the Cikijing River

| Sampling Point | Rainy Season (23-24 March 2022) | | | | | | | | | | |  |
| --- | --- | --- | --- | --- | --- | --- | --- | --- | --- | --- | --- | --- |
|  | **Current Speed ​​(m/s)** | **Temperature (^o^C)** | **DO (mg/L)** | **pH** | **Measurement Time (WIB)** | **Condition of Water Bodies** | **Weather Conditions** | **TDS (mg/L)** | **TSS (mg/L)** | **Conductivity (uS/cm)** | **Salinity (ppt)** | |
| 1 | 0.634 | 25.8 | 1.73 | 8.77 | 10.15 | Cloudy (Lots of household waste) | Sunny Cloudy | 133.6 | 16.7 | 267 | 0.131 | |
| 2 | 0.165 | 27.4 | 1.74 | 8.85 | 11.30 | Blackish cloudy | Sunny Cloudy | 201 | 15.3 | 400 | 0.223 | |
| 3 | 1.62 | 31.5 | 3 | 9.15 | 13.25 | Cloudy Brown | Sunny Cloudy | 1269 | 25.3 | 2550 | 1.31 | |
| 4 | 0.386 | 30.7 | 3.81 | 8.94 | 13.57 | Black | Overcast | 1203 | 23.4 | 2410 | 1.24 | |
| 5 | 0.523 | 29.3 | 3.84 | 8.67 | 14.40 | Black | Drizzling | 1115 | 19.1 | 2230 | 1.12 | |
| 6 | 0.725 | 29 | 3.23 | 8.82 | 15.33 | Black | Bright | 1155 | 20.9 | 2310 | 1.24 | |
| 7 | 0.754 | 30 | 3.62 | 8.89 | 14.49 | Black | Sunny Cloudy | 1420 | 19.1 | 2820 | 1.51 | |
| 8 | 0.775 | 30.2 | 3.22 | 8.87 | 15.16 | Black | Sunny Cloudy | 1362 | 17.6 | 2730 | 1.43 | |
| 9 | 1.12 | 29.4 | 5.13 | 7.76 | 15.55 | Clear Black | Sunny Cloudy | 1338 | 16.7 | 2670 | 1.32 | |
| 10 | 0.674 | 29.5 | 4.81 | 7.85 | 16.17 | Clear Black | Sunny Cloudy | 1298 | 18.1 | 2610 | 1.34 | |
| Maximum | 1.62 | 31.5 | 5.13 | 9.15 | - | - | - | 1420 | 25.3 | 2820 | 1.51 | |
| Minimum | 0.165 | 25.8 | 1.73 | 7.76 | - | - | - | 133.6 | 15.3 | 267 | 0.131 | |
| Average | 0.738 | 29.3 | 3.41 | 8.66 | - | - | - | 1049.5 | 19.2 | 2099.7 | 1.09 | |
| SD | 0.4 | 1.64 | 1.11 | 0.466 | - | - | - | 474.4 | 3.15 | 949.3 | 0.491 | |
| CV | 54.2% | 5.59% | 32.6% | 5.38% | - | - | - | 45.2% | 16.4% | 45.2% | 45.2% | |

**Table 2.** Results of Measurement of Physical-Chemical Parameters in the Dry Season on the Cikijing River

| Sampling Point | Dry Season (29 June 2022) | | | | | | | | | | |  |
| --- | --- | --- | --- | --- | --- | --- | --- | --- | --- | --- | --- | --- |
|  | **Current Speed ​​(m/s)** | **Temperature (^o^C)** | **DO (mg/L)** | **pH** | **Measurement Time (WIB)** | **Condition of Water Bodies** | **Weather Conditions** | **TDS (mg/L)** | **TSS (mg/L)** | **Conductivity (uS/cm)** | **Salinity (ppt)** | |
| 1 | 0.521 | 21.3 | 5.51 | 7.32 | 09.14 | Murky | Bright | 251 | 14.3 | 218 | 0.124 | |
| 2 | 0.332 | 22.5 | 4.32 | 7.44 | 09.36 | Grayish cloudy | Bright | 349 | 16.7 | 296 | 0.131 | |
| 3 | 1.81 | 28.8 | 6.44 | 7.74 | 10.09 | Clear Black | Bright | 3480 | 20.1 | 2950 | 1.51 | |
| 4 | 0.234 | 29 | 6.53 | 7.79 | 10.27 | Black | Bright | 3030 | 16.7 | 2590 | 1.32 | |
| 5 | 0.521 | 18.5 | 6.12 | 7.76 | 10.38 | Brown | Bright | 2860 | 16.7 | 2430 | 1.23 | |
| 6 | 1,141 | 28.9 | 5.73 | 7.74 | 11.08 | Murky Black | Bright | 2980 | 15.3 | 2530 | 1.31 | |
| 7 | 0.412 | 29.4 | 4.22 | 7.81 | 11.23 | Black Brown | Bright | 2840 | 17.7 | 2420 | 1.22 | |
| 8 | 0.324 | 29.4 | 4.41 | 7.74 | 11.45 | Black | Bright | 3470 | 17.2 | 2950 | 1.24 | |
| 9 | 0.633 | 29.5 | 5.34 | 7.69 | 12.09 | Black Brown | Bright | 2720 | 16.2 | 2310 | 1.23 | |
| 10 | 0.414 | 29.5 | 5.91 | 7.69 | 12.22 | Clear Brown | Bright | 2660 | 17.6 | 2250 | 1.24 | |
| Maximum | 1.81 | 29.5 | 6.53 | 7.81 | - | - | - | 3480 | 20.1 | 2950 | 1.51 | |
| Minimum | 0.234 | 18.5 | 4.22 | 7.32 | - | - | - | 251 | 14.3 | 218 | 0.124 | |
| Average | 0.634 | 26.7 | 5.45 | 7.67 | - | - | - | 2464 | 16.9 | 2094.4 | 1.06 | |
| SD | 0.485 | 4.20 | 0.867 | 0.161 | - | - | - | 1174 | 1.54 | 996.9 | 0.497 | |
| CV | 76.5% | 15.7% | 15.9% | 2.10% | - | - | - | 47.6% | 9.14% | 47.6% | 47.1% | |

**Table 3.** Concentrations of Heavy Metals Cr, Cu, Pb, and Zn in Sediment Compartments in the Rainy and Dry Seasons

| Sampling Point | Rainy Season (23-24 March 2022) | | | | Dry Season (29 June 2022) | | | |
| --- | --- | --- | --- | --- | --- | --- | --- | --- |
|  | Cr (mg/kg) | Cu (mg/kg) | Pb (mg/kg) | Zn (mg/kg) | Cr (mg/kg) | Cu (mg/kg) | Pb (mg/kg) | Zn (mg/kg) |
| 1 | 13.7 | 25 | 8.72 | 11578 | 0.051 | 17.1 | 7.85 | 369.2 |
| 2 | 8.3 | 39.8 | 21.0 | 2934.1 | 0.053 | 20.6 | 8.32 | 1513.8 |
| 3 | 14.3 | 31.6 | 10.0 | 56505.9 | 1.95 | 37.9 | 9.04 | 977.7 |
| 4 | 13.9 | 47.6 | 15.7 | 1942.2 | 2.75 | 30.8 | 10.5 | 1877.4 |
| 5 | 9.2 | 66.7 | 8.66 | 957.9 | 3.75 | 30.6 | 10.4 | 1539.0 |
| 6 | 16.5 | 71.3 | 15.5 | 2412.3 | 8 | 27.0 | 10.5 | 61042.2 |
| 7 | 29.1 | 35.1 | 9.85 | 3170.3 | 6.55 | 31.7 | 8.82 | 24397.3 |
| 8 | 18.6 | 33.3 | 8.82 | 3117.5 | 6.8 | 35.0 | 9.7 | 2994.5 |
| 9 | 25.5 | 32.6 | 7.75 | 15785.4 | 0.052 | 29.9 | 7.7 | 13127.1 |
| 10 | 13.3 | 28.8 | 7.75 | 3807.4 | 2.35 | 28.1 | 9.5 | 823.9 |
| Maximum | 29.1 | 71.3 | 21.0 | 56505.9 | 8.0 | 37.9 | 10.5 | 61042.2 |
| Minimum | 8.30 | 25 | 7.75 | 957.9 | 0.051 | 17.1 | 7.72 | 369.2 |
| Average | 16.2 | 41.2 | 11.4 | 10221.1 | 3.23 | 28.9 | 9.23 | 10866.2 |
| SD | 6.62 | 15.9 | 4.47 | 16947 | 2.98 | 6.21 | 1.06 | 19240.8 |
| CV | 40.8% | 38.7% | 39.2% | 165.8% | 92.2% | 21.5% | 11.4% | 177.1% |

**Table 4.** Results of Measurement of Total Organic Content and Sediment Texture

| Sampling Point | Rainy Season (23-24 March 2022) | | | Dry Season (29 June 2022) | | |
| --- | --- | --- | --- | --- | --- | --- |
|  | **Total Organic (%)** | **Sediment Texture** | | **Total Organic (%)** | **Sediment Texture** | |
|  |  | **Sand (%)** | **Mud (Silt & Clay) (%)** |  | **Sand (%)** | **Mud (Silt & Clay) (%)** |
| 1 | 4.64 | 97.3 | 2.74 | 0.852 | 94.0 | 6.04 |
| 2 | 18 | 97.6 | 2.36 | 15.0 | 84.7 | 15.3 |
| 3 | 1.04 | 95.0 | 5.04 | 3 | 91.7 | 8.32 |
| 4 | 23 | 84.2 | 15.8 | 7.91 | 91.2 | 8.83 |
| 5 | 2.67 | 93.8 | 6.18 | 6.62 | 93.4 | 6.64 |
| 6 | 11.7 | 96.4 | 3.65 | 2.63 | 97.9 | 2.12 |
| 7 | 15.2 | 94.3 | 5.74 | 12.9 | 95.6 | 4.43 |
| 8 | 12.4 | 97.4 | 2.6 | 12.4 | 97.4 | 2.58 |
| 9 | 18.8 | 96.5 | 3.46 | 20.7 | 85.9 | 14.1 |
| 10 | 10.8 | 95.7 | 4.28 | 14.9 | 98.2 | 1.79 |
| Maximum | 23.0 | 97.6 | 15.8 | 20.7 | 98.2 | 15.3 |
| Minimum | 1.04 | 84.2 | 2.36 | 0.852 | 84.7 | 1.79 |
| Average | 11.8 | 94.8 | 5.18 | 9.69 | 93 | 7.02 |
| SD | 7.25 | 3.94 | 3.94 | 6.49 | 4.74 | 4.74 |
| CV | 61.4% | 4.2% | 76.1% | 67% | 5.10% | 67.6% |

**Table 5.** Calculation Results of Pollution Index (PI) for Heavy Metal in the Cikijing River

| Pollution Index | | | | | | | | | |
| --- | --- | --- | --- | --- | --- | --- | --- | --- | --- |
| Rainy season | | | | |  | **Dry season** | | | |
| Parameter | Cr | Cu | PB | Zn |  | Cr | Cu | PB | Zn |
| Quality Standard (IV) (mg/L) | 1 | 0.5 | 0.2 | 2 |  | 1 | 0.5 | 0.2 | 2 |
| Sampling Point | Ci/Lij | | | | | | | | |
| Point 1 | 0.007 | 0 | 0 | 0.001 |  | 0 | 0 | 0 | 0.001 |
| Point 2 | 0.011 | 0 | 0 | 0 |  | 0 | 0 | 0 | 0 |
| Point 3 | 0 | 0 | 0.012 | 0.008 |  | 0 | 0 | 0.075 | 0.019 |
| Point 4 | 0 | 0 | 0.010 | 0.384 |  | 0 | 0 | 0.275 | 0.019 |
| Point 5 | 0 | 0 | 0.011 | 0.019 |  | 0 | 0 | 0.090 | 0.020 |
| Point 6 | 0 | 0 | 0.013 | 0.024 |  | 0 | 0 | 0.115 | 0.020 |
| Point 7 | 0 | 0 | 0.017 | 0.001 |  | 0 | 0 | 0.105 | 0.013 |
| Point 8 | 0 | 0 | 0.014 | 0.016 |  | 0 | 0 | 0.180 | 0.014 |
| Point 9 | 0 | 0 | 0.014 | 0.002 |  | 0 | 0 | 0.105 | 0.013 |
| Point 10 | 0 | 0 | 0.014 | 0.029 |  | 0 | 0 | 0.110 | 0.015 |
|  |  |  |  |  |  |  |  |  |  |
| Sampling Point | New Ci/Lij | | | | | | | | |
| Point 1 | 0.007 | 0 | 0 | 0.001 |  | 0 | 0 | 0 | 0.001 |
| Point 2 | 0.011 | 0 | 0 | 0 |  | 0 | 0 | 0 | 0 |
| Point 3 | 0 | 0 | 0.012 | 0.008 |  | 0 | 0 | 0.075 | 0.019 |
| Point 4 | 0 | 0 | 0.010 | 0.384 |  | 0 | 0 | 0.275 | 0.019 |
| Point 5 | 0 | 0 | 0.011 | 0.019 |  | 0 | 0 | 0.090 | 0.020 |
| Point 6 | 0 | 0 | 0.013 | 0.024 |  | 0 | 0 | 0.115 | 0.020 |
| Point 7 | 0 | 0 | 0.017 | 0.001 |  | 0 | 0 | 0.105 | 0.013 |
| Point 8 | 0 | 0 | 0.014 | 0.016 |  | 0 | 0 | 0.180 | 0.014 |
| Point 9 | 0 | 0 | 0.014 | 0.002 |  | 0 | 0 | 0.105 | 0.013 |
| Point 10 | 0 | 0 | 0.014 | 0.029 |  | 0 | 0 | 0.110 | 0.015 |
| Maximum | 0.011 | 0,000 | 0.017 | 0.384 |  | 0,000 | 0,000 | 0.275 | 0.020 |
| Average | 0.002 | 0,000 | 0.011 | 0.048 |  | 0,000 | 0,000 | 0.106 | 0.014 |
| PI | 0.272 | | | |  | 0.196 | | | |
| Category | Meets Quality Standards (Good Water Quality) | | | |  | Meets Quality Standards (Good Water Quality) | | | |

**Table 6.** Contamination Factor (CF) and Pollution Load Index (PLI) Values ​​for Heavy Metals Cr, Cu, Pb, and Zn in Sediment in the Cikijing River During the Rainy Season

| Sampling Point | Contamination Factor (Cf) | | | | Pollution Load Index | | | |  |
| --- | --- | --- | --- | --- | --- | --- | --- | --- | --- |
|  | Cr | Cu | PB | Zn | | PLI value | | Category | |
| 1 | 0.001 | 0.534 | 0.218 | 3.08 | | 0.143 | Contaminated by Several Combinations of Heavy Metals | | |
| 2 | 0.001 | 0.642 | 0.231 | 12.6 | | 0.216 | Contaminated by Several Combinations of Heavy Metals | | |
| 3 | 0.045 | 1,185 | 0.251 | 8.15 | | 0.576 | Contaminated by Several Combinations of Heavy Metals | | |
| 4 | 0.064 | 0.964 | 0.291 | 15.6 | | 0.728 | Contaminated by Several Combinations of Heavy Metals | | |
| 5 | 0.087 | 0.955 | 0.288 | 12.8 | | 0.745 | Not Polluted by Some Combinations of Heavy Metals | | |
| 6 | 0.186 | 0.843 | 0.293 | 508.7 | | 2.20 | Contaminated by Several Combinations of Heavy Metals | | |
| 7 | 0.152 | 0.991 | 0.245 | 203.3 | | 1.66 | Contaminated by Several Combinations of Heavy Metals | | |
| 8 | 0.158 | 1,094 | 0.269 | 25.0 | | 1.04 | Contaminated by Several Combinations of Heavy Metals | | |
| 9 | 0.001 | 0.934 | 0.214 | 109.4 | | 0.399 | Contaminated by Several Combinations of Heavy Metals | | |
| 10 | 0.055 | 0.879 | 0.263 | 6.87 | | 0.543 | Contaminated by Several Combinations of Heavy Metals | | |
| Maximum | 0.677 | 2.23 | 0.583 | 470.9 | | 2.56 | - | | |
| Minimum | 0.193 | 0.782 | 0.215 | 7.98 | | 0.961 | - | | |
| SD | 0.154 | 0.497 | 0.124 | 141.2 | | 0.458 | - | | |
| CV | 40.8% | 38.7% | 39.2% | 165.8% | | 29.6% | - | | |
| Average | 0.075 | 0.902 | 0.256 | 90.6 | | 0.824 | **Contaminated by Several Combinations of Heavy Metals** | | |
| Category | Low Polluted | Moderately Polluted | Low Polluted | Very Polluted | |  | |  | |

**Table 7.** Contamination Factor (CF) and Pollution Load Index (PLI) Values ​​for Heavy Metals Cr, Cu, Pb, and Zn in Sediments in the Cikijing River During the Dry Season

| Sampling Point | Contamination Factor (Cf) | | | | Pollution Load Index | | | |  |
| --- | --- | --- | --- | --- | --- | --- | --- | --- | --- |
|  | Cr | Cu | PB | Zn | | PLI value | | Category | |
| 1 | 0.00 | 0.53 | 0.22 | 3.08 | | 0.14 | Not Polluted by Some Combinations of Heavy Metals | | |
| 2 | 0.00 | 0.64 | 0.23 | 12.61 | | 0.22 | Not Polluted by Some Combinations of Heavy Metals | | |
| 3 | 0.05 | 1.18 | 0.25 | 8.15 | | 0.58 | Not Polluted by Some Combinations of Heavy Metals | | |
| 4 | 0.06 | 0.96 | 0.29 | 15.64 | | 0.73 | Not Polluted by Some Combinations of Heavy Metals | | |
| 5 | 0.09 | 0.96 | 0.29 | 12.83 | | 0.74 | Not Polluted by Some Combinations of Heavy Metals | | |
| 6 | 0.19 | 0.84 | 0.29 | 508.69 | | 2.20 | Contaminated by Several Combinations of Heavy Metals | | |
| 7 | 0.15 | 0.99 | 0.24 | 203.31 | | 1.66 | Contaminated by Several Combinations of Heavy Metals | | |
| 8 | 0.16 | 1.09 | 0.27 | 24.95 | | 1.04 | Not Polluted by Some Combinations of Heavy Metals | | |
| 9 | 0.00 | 0.93 | 0.21 | 109.39 | | 0.40 | Not Polluted by Some Combinations of Heavy Metals | | |
| 10 | 0.05 | 0.88 | 0.26 | 6.87 | | 0.54 | Not Polluted by Some Combinations of Heavy Metals | | |
| Maximum | 0.186 | 1.18 | 0.293 | 508.7 | | 2.20 | - | | |
| Minimum | 0.001 | 0.534 | 0.214 | 3.08 | | 0.143 | - | | |
| SD | 0.069 | 0.194 | 0.029 | 160.3 | | 0.650 | - | | |
| CV | 92.2% | 21.5% | 11.5% | 177.1% | | 78.8% | - | | |
| Average | 0.08 | 0.90 | 0.26 | 90.55 | | 0.82 | **Not Polluted by Some Combinations of Heavy Metals** | | |
| Category | Low Polluted | Low Polluted | Low Polluted | Very Polluted | |  | |  | |

**Table 8.** Geoaccumulation Index Results for Heavy Metals Cr, Cu, Pb, and Zn in Sediments During the Rainy and Dry Seasons

| Sampling Point | Rainy Season Geoaccumulation Index (Igeo). | | | | Dry Season Geoaccumulation Index (Igeo). | | | |
| --- | --- | --- | --- | --- | --- | --- | --- | --- |
|  | **Cr** | **Cu** | **PB** | **Zn** | **Cr** | **Cu** | **PB** | **Zn** |
| 1 | -3.31 | -1.22 | -1.98 | 6.01 | -11.4 | -1.77 | -2.14 | 1.04 |
| 2 | -4.02 | -0.556 | -0.717 | 4.03 | -11.4 | -1.51 | -2.05 | 3.07 |
| 3 | -3.24 | -0.889 | -1.78 | 8.29 | -6.11 | -0.626 | -1.93 | 2.44 |
| 4 | -3.28 | -0.297 | -1.13 | 3.43 | -5.62 | -0.924 | -1.72 | 3.38 |
| 5 | -3.88 | 0.189 | -1.99 | 2.41 | -5.17 | -0.936 | -1.74 | 3.10 |
| 6 | -3.04 | 0.285 | -1.15 | 3.74 | -4.08 | -1.12 | -1.71 | 8.41 |
| 7 | -2.21 | -0.738 | -1.81 | 4.14 | -4.37 | -0.884 | -1.97 | 7.08 |
| 8 | -2.86 | -0.813 | -1.97 | 4.11 | -4.31 | -0.740 | -1.83 | 4.06 |
| 9 | -2.40 | -0.845 | -2.15 | 6.45 | -11.4 | -0.970 | -2.16 | 6.19 |
| 10 | -3.34 | -1.02 | -2.15 | 4.40 | -5.84 | -1.06 | -1.86 | 2.19 |
| Maximum | -2.21 | 0.285 | -0.717 | 8.29 | -4.08 | -0.626 | -1.71 | 8.41 |
| Minimum | -4.02 | -1.22 | -2.15 | 2.41 | -11.4 | -1.77 | -2.16 | 1.04 |
| SD | 0.569 | 0.503 | 0.500 | 1.72 | 3.13 | 0.346 | 0.168 | 2.36 |
| CV | 18.0% | 85.1% | 29.7% | 36.6% | 44.9% | 32.9% | 8.8% | 57.6% |
| Average | -3.16 | -0.59 | -1.68 | 4.70 | -6.97 | -1.05 | -1.91 | 4.10 |
| Category | Not Polluted | Not Polluted | Not Polluted | Heavily polluted/severe to very heavy | Not Polluted | Not Polluted | Not Polluted | Heavily polluted/severe to very heavy |
