# Supplementary material for: Heavy metals in water and sediment of Cikijing River, Rancaekek District, West Java: Contamination distribution and ecological risk assessment
Source: PLoS One. 2024 Apr 17;19(4):e0294642. doi: 10.1371/journal.pone.0294642 (PMC11023565; doi:10.1371/journal.pone.0294642)
Supplement: Attachment — Submitted filename: PlosOne-PONE-D-23-01059.docx [file pone.0294642.s002.docx]

COMMENTS ON MANUSCRIPT ID: PONE-D-23-01059

The manuscript presents “Analysis of Water Quality and Potential Ecological Risk Based on Heavy Metal Content (Cr, Cu, Pb, and Zn) in the Cikijing River, Rancaekek District, West Java”, which is remarkable. The subject addressed is within the scope of the journal.

Overall, the study is rich in data and well discussed. However, the manuscript, in its present form, contains several weaknesses. The language is not bad but best polished by native English speakers. However, the authors need to consider the following questions, some of which are clearly flawed and may be difficult to meet publication standards without addressing them well. Some inconsistencies and minor errors that needed attention are:

For readers to quickly catch your contribution, it would be better to highlight major difficulties and challenges, and your original achievements to overcome them, in a clearer way in abstract and introduction. Abstract should be informative and include the main findings. It would be proper if the authors should link the concentration of the heavy metals in the study area to the geology or other sources in the area.

It would be appropriate if a comparison table was made with the values found around the world. It is better to calculate the overall average values of each element in the present study while comparing it with other studies or please provide the range for all the compared studies.

Please check the legends on the figures and/or tables for completeness, such as if values are means, SD or SE, n = replicates, and also the details and explanations of any statistical labels.

Two sampling times were set up as rainy and dry seasons in 2022. How many sampling carried out during the study? How many stations did you set up? How many samples in total?

Were the samples dried in air temperature?? Please specify the temperature and time period for the drying process.

Did you use grinder and sieve prior to digestion of sediment samples?

You might present the recovery results of the certified reference material in a separate table to show your % more clearly.

Did surrogate be used in the extraction procedure? What's that?

The interaction between shale and heavy metal is not given as exactly correct. The interaction between sediment and heavy metal is not given as exactly correct. It should be re-explained in detail and given correctly. For example the age of the sediment is important determinant.

The authors should also compare the results with the standard acceptable levels established by the Minister of Environment Number 115 of 2003. What are the other feasible alternatives? What are the advantages of adopting these particular standards over others in this case? How will this affect the results? More details should be furnished.

In general, figures and tables are quite simple and carelessly prepared. Improvements are required for the tables and figs. For example, Figure 1. " Map of Sampling Points The Cikijing River" is insufficient to show the study area. In the first place, the country where the study was conducted and its place in the world is not clearly shown.

You wrote "mg kg^-1^" in the tables and "mg/kg" in the figures. "mg kg^-1^" or "mg/kg" please be consistent. Also, use either “wet w” or “dry w”.

The discussion section in the present form is relatively weak and should be strengthened with more details and justifications.

Please check for some typo/grammar (few places).

In the conclusion section, the limitations of this study, suggested improvements of this work and future directions should be highlighted.

Moreover, the manuscript could be substantially improved by relying and citing more on recent literatures about contemporary real-life case studies on sustainability and/or metal accumulations such as the followings:

<https://doi.org/10.1016/j.iswcr.2018.09.001>

<https://doi.org/10.1080/15275922.2020.1728433>

<https://doi.org/10.1007/s10661-022-10490-1>

<https://doi.org/10.1111/j.1749-7345.2002.tb00499.x>

Mention about limitations of your study if you have any in conclusion section
