# Supplementary material for: Heavy metals in water and sediment of Cikijing River, Rancaekek District, West Java: Contamination distribution and ecological risk assessment
Source: PLoS One. 2024 Apr 17;19(4):e0294642. doi: 10.1371/journal.pone.0294642 (PMC11023565; doi:10.1371/journal.pone.0294642)
Supplement: Attachment — Submitted filename: Comments to Reviewer_PONE.docx [file pone.0294642.s003.docx]

| Reviewer 1 | | Comments or Revision |
| --- | --- | --- |
| 1 | The limitations of this study, suggested improvements of this work should be highlighted. | Limitation of this study has been added in the manuscript |
| 2 | Manuscript could be substantially improved by relying and citing more on recent literatures about contemporary real-life case studies on sustainability and/or metal accumulations such as the followings:  https://doi.org/10.1016/j.iswcr.2018.09.001  https://doi.org/10.1080/15275922.2020.1728433  https://doi.org/10.1007/s10661-022-10490-1 | Those papers have been discussed and cited in Introduction section especially at paragraph 3 and 4 |
| 3 | The authors should link the concentration of the heavy metals in the study area to the geology | Have been added in Introduction (paragraph 8) |
| 4 | It would be appropriate if a comparison table was made with the values found around the world. | Have been added in Table 12 |
| 5 | Please check the legends on the figures and/or tables for completeness | The legend in the figures and/or tables has been corrected and added |
| 6 | Two sampling times were set up as rainy and dry seasons in 2022. | A full explanation have been added in the materials and methods section |
| 7 | Specify the temperature and time period for the drying process. | A full explanation has been added in the Data Collection section |
| 8 | Did you use grinder and sieve prior to digestion of sediment samples? | We use sieve prior identification of sediment texture |
| 9 | the recovery results of the certified reference material in a separate table to show your % more clearly | The results of measurements of sediment texture at sampling locations both during the rainy and dry seasons has been added in Table 13 |
| 10 | The interaction between shale, sediment, and heavy metal should be re-explained in detail and given correctly. | thank you very much for the suggestion, the interaction between heavy metals and shale, sediment is based on field data and processed using the IBM SPSS Statistics application |
| 11 | The authors should also compare the results with the standard acceptable levels established by the Minister of Environment Number 115 of 2003. | Have been added in Results and Discussion (Paragraph 2) |
| 12 | Improvements are required for the tables and figs. | Thanks for the suggestion, the figures and tables have been fixed |

| Reviewer 2 | | Comments or Revision |
| --- | --- | --- |
| 1 | Author must be change the title of this paper and need to include components like water and sediment. | The title of this paper has been revised with include components of water and sediment  The title become:  “Heavy Metals in Water and Sediment of Cikijing River, Rancaekek District, West Java: Contamination Distribution and Ecological Risk Assessment” |
| 2 | Author must rewrite this section  Why HMs harmful environment? | Narration about HMs have been added in Introduction (paragraph 2) |
|  | Describe Literature (HMs) in global as well as authors country | Have been added in Introduction (paragraph 6) |
|  | How it is related in food chain? | Have been added in Introduction (paragraph 5) |
|  | What is the significant of this studies? | Have been added in Introduction (Last paragraph) |
| 3 | Author can follow these papers:  a. https://www.tandfonline.com/doi/full/10.1080/15569543.2021.1891936  b. https://doi.org/10.1016/j.scitotenv.2020.144637  c. https://link.springer.com/article/10.1007/s11356-021-15353-9  d. https://doi.org/10.1016/j.ijsrc.2021.09.002 | Those papers have been discussed and cited in Introduction section especially at paragraph 5 |
| 4 | PCA and cluster analysis must be included for source identification (MM) | Actually, in this study, the location itself which is Cikijing River is specific location or area that consist of several industries, especially textile industry. So we consider, source identification not really have to be done, because this location is a specific location with specific type of pollution (industries pollutants) |
| 5 | I think author should give advice for government and stakeholders and so on. | Advice for government have been added into the manuscript |
| 6 | The abstract should stand alone with focused results. | Have been added in Abstract (Sentences 4-7) |
| 7 | Study area must be described significantly why authors select this area | Have been added in Location and Time of Sampling (paragraph 2) |
| 8 | Authors include the station id in the map | Have been added in Figure 1 |
| 9 | Author can describe all pollution in single table to reduce the paper length and summarize it. | We apologize because each assessment method has different parameters and subjects so it is less effective to combine them into a single table |
| 10 | all of the figures are totally blur and must be redraw | Thanks for the suggestion, the figures has been fixed |
| 11 | Comparison table must be needed to compare their studies with others studies in the globe | A comparison of studies with research in various parts of the world has been included in the discussion and Table No.12 |
