# Supplementary material for: Heavy metals in water and sediment of Cikijing River, Rancaekek District, West Java: Contamination distribution and ecological risk assessment
Source: PLoS One. 2024 Apr 17;19(4):e0294642. doi: 10.1371/journal.pone.0294642 (PMC11023565; doi:10.1371/journal.pone.0294642)
Supplement: Attachment — Submitted filename: 2Comments to Reviewer PONE (1).docx [file pone.0294642.s004.docx]

Comments to Reviewer

| No | Comments | Response from Author |
| --- | --- | --- |
| 1 | Adverse health effects of analyzed elements must be presented in the introduction section.  In so doing, it is suggested that the following articles be used as a reference:  i) Marine Pollution Bulletin, 123(1-2): 34-38 (2017). 13 | Has been added in Introduction section |
| 2 | ii) Environmental Science and Pollution Research, 27(12): 13301-13314 (2020). | Has been added in Introduction section |
| 3 | Bulletin of Environmental Contamination and Toxicology, 109(6): 1142-1149 (2022 | Has been added in Introduction section |
| 4 | The results of 'one way ANOVA' and 'one-sample t-test' analysis must be presented and discussed to comparing the mean levels of studied PTEs between the sampling sites and also between the mean levels of PTEs and maximum permissible concentrations (MPCs) | One way ANOVA in this research is used to determine whether parameter data is homogeneous or not to choose the correlation method used. It has been added in the discussion section. |
| 5 | The correlation matrix between the mean content of analyzed elements and physicochemical parameters of sediment and water samples | It has been added in the discussion section. |
| 6 | The reference values of PTEs in Indonesia | Potential Toxic Elements for the heavy metals analyzed have not yet been regulated in Indonesia |
| 7 | The values of EF and Nemrow indices must be presented and clearly discussed | Based on other journal references, the EF and Newrow methods cannot be used in this analysis because the three methods used, namely Igeo, PLI, and PERI, can already present heavy metal contamination in ecology. |
| 8 | The values of SD and CV(%) of analyzed elements must be presented. Point sign must be used as decimal. Improved number writing. | It has been corrected and added to the manuscript |
